# Shared book reading to promote mental well-being among young people with and without Down syndrome: a pilot dyadic randomized controlled trial

**DOI:** 10.3389/fpubh.2025.1604241

**Published:** 2025-10-29

**Authors:** Xiaoyi Huang, Iman Long, Wenjie Zhang, Jikun Xu, Robert David Smith

**Affiliations:** Unit of Psychiatry, Department of Public Health and Medicinal Administration, Faculty of Health Sciences, Institute of Translational Medicine, University of Macau, Macao SAR, China

**Keywords:** shared book reading, Down syndrome, young people, mental well-being, randomized controlled trial, protocol, pilot study

## Abstract

**Background:**

With societal progress and a deepening understanding of Down syndrome (DS), research focus has shifted toward improving the quality of life and education for youth with DS. This study aims to determine the feasibility and estimate the preliminary effectiveness of a dyadic shared book reading program on the health-related quality of life of youth with DS (primary outcome), the mental wellbeing of university students (secondary outcome), and actor–partner outcomes to inform a future definitive trial.

**Methods:**

This study is an 8-week pilot dyadic randomized controlled trial (RCT) comparing a shared book reading intervention to a minimal activity control. The study will then continue for 12 months as a single-arm cohort study. Young people with DS and university students will be recruited, formed into dyads, and then randomized to either the intervention or control group. The intervention involves pairing a young person with DS with a university student for a weekly, 1-h shared book reading session. The control group will be provided with three books to read at their leisure over the 8 weeks. The primary outcome is the health-related quality of life of young people with DS, measured using the Pediatric Quality of Life Inventory (PedsQL 4.0). Secondary outcomes include the Engagement, Perseverance, Optimism, Connectedness, and Happiness (EPOCH) scale for measuring wellbeing. The Friendship Quality Questionnaire (FQQ) will be used to measure the dyad's level of friendship in the intervention group at 8 weeks. Measurements will be taken at baseline (T0) and at 8 weeks (T1). After 8 weeks, all participants will be offered the opportunity to continue in the study, joining the weekly shared book reading intervention; outcome measures will then be assessed at 6-month (T2) and 12-month (T3) follow-ups. Mixed linear regression models will be used to compare the intervention and control groups at 8 weeks. For the 6-month and 12-month follow-ups, change scores from baseline will be analyzed to test for potential long-term effect.

**Discussion:**

This study focuses on the mental wellbeing of young people with DS and university students by promoting psychological health through shared book reading activities.

**Anticipated results:**

It is expected that the shared book reading activities will improve the mental wellbeing of both young people with DS and university students; however, due to the pilot study's sample size, this trial may not detect effectiveness at a level of statistical significance.

**Clinical Trial Registration:**

Clinicaltrials.gov, identifier NCT06813625.

## Introduction

Down syndrome (DS) is a congenital genetic disorder caused by chromosomal abnormalities (1). This condition can impact an individual's intellectual and physical development, leading to challenges in managing daily life and learning. Currently, the global incidence of DS is approximately 14 per 10,000 live births. This rate may vary across different sociocultural contexts due to disparities in access to genetic screening and attitudes toward pregnancy termination (2). In the United States, about 6,000 babies are born with DS each year, corresponding to an incidence of approximately 1 in every 700 births (3). In the UK, the prevalence is 12.6 per 10,000 live births, with around 750 babies born with DS annually (4). The incidence of DS among live births in China is notably lower, at 3.05 per 10,000 live births (5).

The impact of DS on individuals and their families is multifaceted. Individuals with DS often experience challenges in cognitive development, physical growth, and facial characteristic (6). These challenges can affect learning, language, and memory, and are also associated with increased health risks such as congenital heart disease, intestinal malformations, and vision or hearing impairments (7). Such physical and cognitive differences may impede the social development of a child with DS, who often struggles to form and maintain peer friendships. This can limit social wellbeing and emotional development, further reducing opportunities for social participation (8). A lack of social interaction has been strongly linked to lower quality of life, which is particularly evident in the DS population. Social engagement is crucial for quality of life in people with DS, as it not only provides a sense of fulfillment but also supports relationship-building and involvement in sports, hobbies, and other meaningful activities (9). Improving social engagement and quality of life for individuals with DS requires active support and acceptance from family, the community, and society as a whole.

Individuals with DS often demonstrate relative strengths in non-verbal learning, visual processing, and social skills, despite cognitive delays (10, 11). These strengths can be leveraged through social interventions such as shared book reading to promate engagement and learning. Shared book reading has been shown to positively influence the mental wellbeing of young people. Through reading and interactive discussion, youth can develop a deeper understanding of mental wellbeing concepts and increase their awareness of related issues. Additionally, shared book reading can stimulate enthusiasm for social interaction and make reading a more enjoyable experience (12). This activity may not only improve social participation and quality of life but also help young people better cope with challenges and achieve personal growth and development (13).

Although this is the first shared book reading intervention involving young people with DS and university students in Macau, previous studies on shared book reading have indicated positive outcomes. Research by Sun et al. (14) demonstrated that shared book reading can significantly improve emotional regulation and social skills in children, particularly among those with developmental disabilities. Similarly, another study found that shared book positively influences children's language development and mental wellbeing (15). Shared book reading involves interactive, paired reading accompanied by discussion and reflection. Although these studies focused primarily on school-aged children or individuals with other developmental conditions rather than specifically on DS, they suggest that shared reading can contribute to emotional and social benefits. Our study seeks to expand this evidence to young people with DS- a population that has not been studied in the context of such peer-interaction interventions. We hypothesize that shared book reading will not only improve mental wellbeing but may also enhance quality of life by promoting social engagement and emotional support, as indicated by existing literature.

Shared book reading may serve as an effective educational tool to help young people with DS improve language, cognitive, and social skills (16). By reading with others, young individuals can gain exposure to new knowledge and information, broaden their perspectives, and build self-confidence. Additionally, shared book reading can facilitate social and emotional exchanges among young people, their families, and peers, promoting mutual understanding and respect while contributing to a more inclusive and supportive environment (17). However, to date, there is a lack of research on shared book reading interventions that pair young people with DS with typically developing peers. This study aims to address that gap.

This study aims to pilot test the feasibility and evaluate the preliminary effects of a shared book reading intervention on the quality of life of young people with DS. The study also includes two secondary objectives:

**Secondary objective 1:** To examine whether shared book reading with young people with DS improves the mental wellbeing of participating university students.

**Secondary objective 2:** To investigate the actor–partner interdependence between young people with DS and their student partners in terms of quality of life and mental wellbeing outcomes.

## Methods

**Figure 1:**
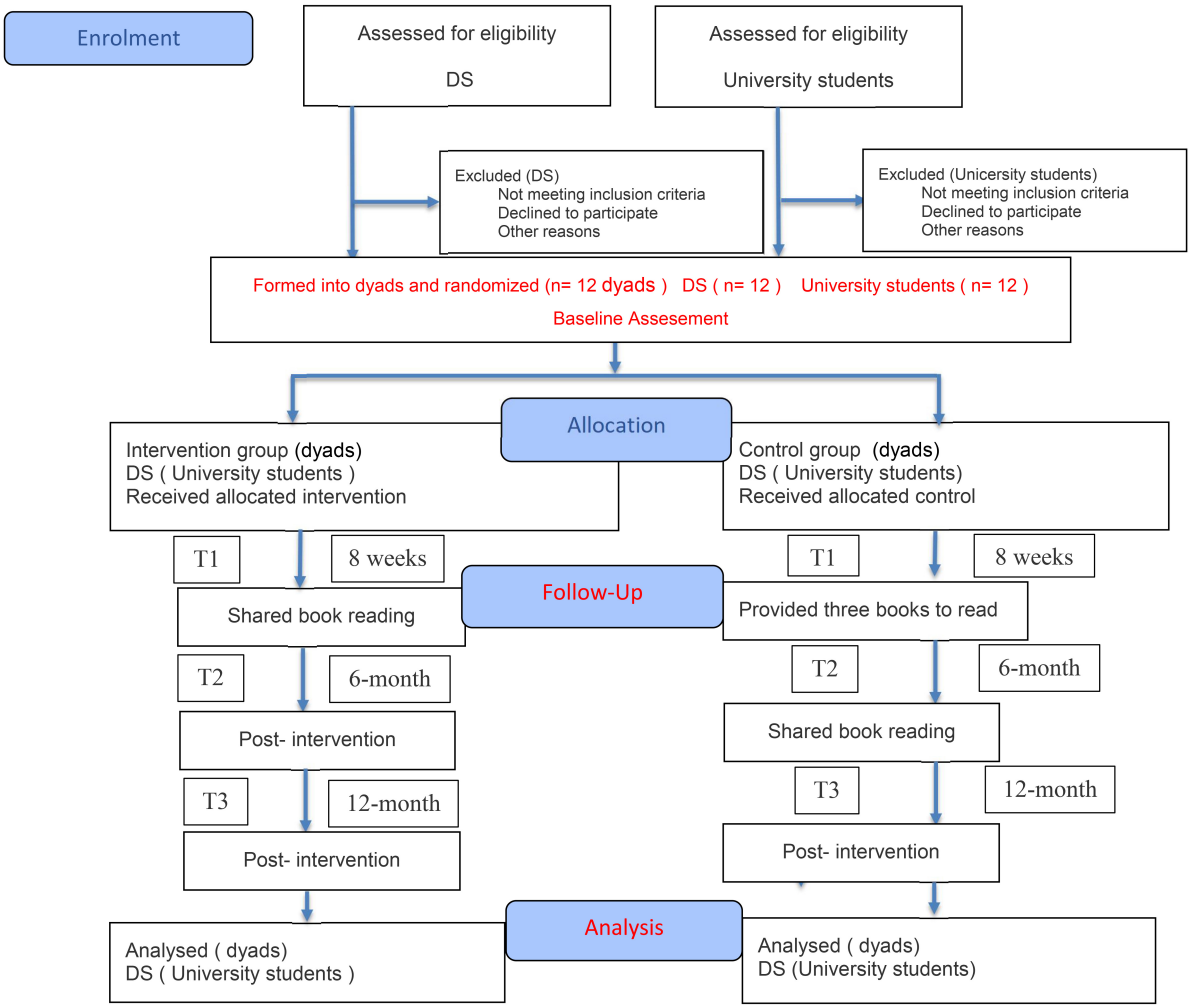
CONSORT 2025 flow diagram.

The study protocol has been developed and reported in accordance with the SPIRIT guidelines and follows the CONSORT 2025 flow diagram (Figure 1) (18).

### Patient and public involvement (PPI)

In recent years, PPI has gained widespread recognition in health-related research across many countries. However, adoption of this approach has been limited to date in China (19). PPI was incorporated into this study to ensure the relevance and appropriateness of the findings for young people with DS and their families. Throughout the development of the study, we involved young people, parents, caregivers, and public members in key discussions and decisions. The study was initially conceived and developed through PPI. The researchers, in collaboration with the Macau Down Syndrome Association (MDSA) organized two PPI meetings during the design phase of this study. The first meeting included researchers, young people with DS, their parents or guardians, and university students. During these sessions, researchers presented the concept of a shared book reading intervention involving university students and young people with DS, which received broad support. Several refinements were made based on PPI feedback regarding recruitment strategies, intervention length and content, outcome measures, and the location of intervention activities. A second meeting with MDSA representatives and parents/guardians led to further agreements on the management and scheduling of intervention activities. For example, weekends were preferred due to school commitments. In this meeting, PPI members also recommended incorporating engaging elements into the intervention to attract and sustain participant interest.

Through our co-design process, the PPI group played an active role in shaping the intervention, particularly in selecting and reviewing the reading materials, refining the intervention design, and defining the role of the supporters. The PPI group also served as key collaborators in identifying and selecting outcome measures for the study. The group will continue to provide mutual support, facilitated by the research team and the PPI lead. The PPI Advisory Panel will maintain close collaboration with the research team throughout the duration of the study.

### Study design

This study will be a pilot pragmatic parallel-group dyadic randomized controlled trial (RCT) comparing a shared book reading intervention (young people with DS paired with university student partners) to a minimal-activity control group.

All individuals with DS recruited into the study will be randomly paired with a university student to form a dyad. Depending on recruitment numbers, if more university students are enrolled than young people with DS, some dyads may consist of one participant with DS and two students (i.e., a 1:2 pairing). Each dyad will be randomized 1:1 to either the intervention group or the control group. Recruitment will take place over a 12-month period, with each participant involved in their assigned condition for 8 weeks. After this period, participants in the control group will also be offered the intervention. The primary outcome will be assessed at 8 weeks post- randomization. An extended phase will allow for exploration of long-term effects and sustainability, with outcomes measured at 6 and 12 months to inform future trial design.

### Setting

Young people with DS will be recruited from community settings in Macau in close collaboration with the MDSA. University students will be recruited from universities in Macau.

### Participants

Inclusion criteria for participants with DS are as follows: (1) diagnosis of DS; (2) aged 10–20 years; (3) ability to participate in the intervention and assessments either independently or with caregiver support. Potential participants will be approached through DS community centers and social media advertisements. The age range was selected based on discussion and agreement with the PPI group, which included members of the Macau Down Syndrome Association with experience organizing social activities for this population. Based on their prior experience, the 10–20 years are group was deemed most appropriate.

The inclusion criteria for university student participants are as follows: (1) age 18 years or older; (2) ability to read and understand books in English or Chinese; (3) ability to communicate in English, Mandarin, or Cantonese; (4) absence of severe visual or reading impairments that would affect reading ability. Exclusion criteria include: current hospitalization; severe comorbidities (e.g., uncontrolled epilepsy) or inability to participate even with caregiver support; inability to participate due to severe visual or speech impairments without an available caregiver to assist); or concurrent participation in another clinical study that could interfere with this intervention or its assessments.

While the age range for participants with DS is broad, the intervention focuses on fostering mentorship and social connection. The university students will act as supportive partners, adapting their engagement strategies to the developmental level of their assigned participant. All participants (or their legal guardians, for participants with DS) will provide informed consent after a full explanation of the study procedures, time commitment, and their rights (including the voluntary nature of participation and the right to withdraw at any time). We will ensure they have ample time to ask questions and consider participation before signing the consent form. For participants with DS who are minors or require guardian support, consent will be obtained from their caregivers (legal representatives) in addition to assent from the participant. We will use easy-to-understand language and visual aids to explain the study to ensure that participants with DS and their caregivers fully understand the study's purpose, procedures, and potention risks. All participants will be given sufficient time to consider participation. Young people with DS and university student participants in both groups will receive a shopping voucher worth 160MOP (approximately $20USD) upon completion of the 8-week follow-up assessment.

### Forming dyads

We will randomly pair each young person with DS with at least one university student to form dyads and facilitate shared reading experiences. This pairing is designed to encourage social connection and friendship development between the partners.

### Randomization and allocation

Participants within their dyads will be randomized (1:1) to either the intervention or control group. A computer-generated randomization list (using random block sizes of 2 and 4) will be prepared at the University of Macau, and allocation will occur after baseline data collection. An external statistician not involved in the study will perform the allocation centrally using this list. Researchers conducting the baseline and follow-up assessments will be blinded to group allocation.

### Shared book reading intervention

The shared book reading intervention will be led by an intervention leader, a doctoral research student who is a registered nurse. The intervention leader will be assisted by facilitators who will receive 1-h training in DS-adapted communication strategies (e.g., visual supports, paced questioning) and engagement techniques (e.g., dialogic reading, sensory accommodations). Sessions will comprise: (i) a 10-min icebreaker involving a musical chairs adaptation (flower-passing with music, followed by sharing a favorite word from previous sessions) and facial expression imitation games to build rapport; (ii) a 30-min shared reading using PPI-approved books, with adaptations for non-verbal participants (yes/no questions) and attention challenges (10-min reading blocks with stretch breaks); and (iii) a 10-min reflection using visual prompts (emoji cards for “favorite part” discussions) and icon-based logbook entries. This training will be delivered by the intervention leader. Weekly facilitator reports will document implementation challenges (e.g., participant disengagement) for iterative problem-solving.

Facilitators will maintain participant's engagement, and caregivers of young people with DS are welcome to attend. To minimize bias, facilitators will follow a standardized manual (Supplementary File 1: Intervention Facilitator Manual). Session fidelity across all dyads will be assessed using checklists (Supplementary File 2: Fidelity Checklist and Scoring Criteria) completed by the intervention leader. Each session will conclude with a brief reflection period during which participants discuss what they have read and plan for the next session. Weekly reminders will be sent to families and students regarding session times. Attendance will be incentivized through a sticker system, with participants collecting stickers in a provided scrapbook.

As suggested during PPI meetings, we will introduce a “Growth Record” logbook (Supplementary File 3: Participant Growth Logbook Template) for each participant with DS. This personal logbook allows the young people (with caregiver assistance as needed) to record each session's reading activities—such as books read, new vocabulary, and personal reflections or progress. Caregivers are encouraged to help maintain the log, which supports tracking the child's development and sustains engagement with the intervention at home. The logbook is optional and adaptable for all ages. Caregivers will receive guidance on supporting home reading activities, though no formal home practice is required. The growth log also serves as a bridge between the child, family, and student volunteer, enabling sharing of entries or achievements to foster communication and a sense of accomplishment. Families are encouraged to continue using the log after the study to support sustained reading habits. This logbook concept emerged from PPI feedback aimed at enhancing participant engagement.

### Control group

Participants in the waitlist control group will be informed that they must wait 8 weeks before beginning the shared book reading sessions. Dyads in the control group will not be introduced during this 8-week period; participants with DS and students in this arm will only meet after the 8-week follow-up, when the intervention begins. To maintain engagement among youth with DS in the control group, they will be provided with three books to read independently or with their family during the waiting period. After the 8-week wait, control group participants will be invited to join the shared reading sessions. At that point, each young people with DS in the control group will be introduced to their student partner and may then participate fully in the weekly sessions. This approach ensures that while the control group receives books, they do not engage in dyadic shared reading until after the 8-week waitlist period. This design is methodologically critical, aligns with PPI group consensus that all participants eventually receive the intervention, and fulfills ethical requirements.

### Outcome measures

Outcome measures will be collected at baseline (T0) and 8 weeks post-randomization (T1) for both the intervention and control groups. Following the initial 8-week RCT phase, all participants (including those from the waitlist control group) will receive the intervention; additional follow-ups will be conducted at 6 months (T2) and 12 months (T3) post-randomization in this single-arm extension phase. For the intervention group, outcomes for participants with DS will be collected at the end of the final session through researcher-administered interviews. For the control group, 8-week outcomes for participants with DS will be collected via researcher-conducted telephone interviews. University student participants in both groups will complete their outcome measures through an online survey, with reminder prompts sent after 1 and 2 days if not completed.

At T1, dyads in the intervention group will complete one additional measure (assessing the dyad's overall friendship) that will not be administered to the waitlist control group; all other outcome measurements are identical for both groups. The primary outcome of this study is the health-related quality of life of young people with DS at 8 weeks (T1), measured using the Pediatric Quality of Life Inventory (PedsQL)4.0. Secondary outcomes include the mental wellbeing of the student partners (assessed using the EPOCH scale) and the quality of the friendship within the dyad (assessed via the Friendship Quality Questionnaire, FQQ). Additionally, caregivers of participants with DS will complete a Global Perceived Effect (GPE) scale to evaluate their perception of change in the quality of life of the participant with DS.

### Baseline characteristics

Baseline measurements will be recorded prior to randomization. Participant characteristics, including age, sex, height (cm), weight (kg), ethnicity, language, type of residence, and education level, will be collected. Caregiver information will also be recorded, including the caregiver's relationship to the participant and caregiver age. All outcome data collection points throughout the study timeline are presented in Table 1.

**Table 1:** Data collection components and collection timeline.

**Construct**	**Outcome measure**	**Group**	**Baseline (T0)**	**8 weeks (T1)**	**6 months (T2)**	**12 months (T3)**
Socio-demographics		All participants	√	–	–	–
Health-related QoL	PedsQL 4.0	DS participants	√	√ (I+C)	√	√
		University students	√	√ (I+C)	√	√
Mental wellbeing	EPOCH	University students	√	√ (I+C)	√	√
Global perceived effect	GPE	DS caregivers	–	√ (I+C)	√	√
Friendship quality	FQQ	Intervention dyads only	–	√ (I)	√	√

DS, Young people with Down syndrome; I, Intervention group; C, Control group; PEDSQL 4.0, Pediatric Quality of Life Inventory version 4.0; EPOCH, Engagement, Perseverance, Optimism, Connectedness, and Happiness; GPE, The Global Perceived Effects scale; FQQ, Friendship Quality Questionnaire.

#### Health-related quality of life

Health-related quality of life for young people with DS will be measured using the PedsQL 4.0. The Pediatric Quality of Life Inventory (PedsQL) is a widely used tool to assess quality of life in children and adolescents aged 2–18 years with acute or chronic health conditions, including DS (20). For participants over 18 years old, the adult version of the PedsQL will be used where appropriate, or caregiver proxy reports will be obtained. The PedsQL 4.0 covers domains of physical functioning, emotional wellbeing, social interaction, and school functioning, and also provides a total score. Higher scores indicate better quality of life. This instrument has demonstrated good psychometric properties in young people, with internal consistency (Cronbach's α) ranging from 0.66 to 0.93 (21).

Caregivers of participants with DS will complete the GPE scale for QOL at T1 to provide an external assessment of change in the child's QOL. For analysis, responses of “very much improved” or “much improved” will be categorized as a clinically important improvement, while responses of “a little improved,” “no change,” or “worse” will be considered as no substantial improvement (22). GPE responses will be analyzed as an ordinal scale, with “worse” reported separately to assess potential deterioration.

#### Mental wellbeing

The EPOCH Measure of Adolescent Wellbeing is a scale used to assess positive psychological traits in young people (23). It consists of five dimensions: Engagement, Perseverance, Optimism, Connectedness, and Happiness. The EPOCH includes 20 items (4 per dimension) and was developed as a brief measure of positive youth psychological traits that may promote wellbeing and physical health (24).

#### Friendship quality

Friendship quality will be assessed using the Friendship Quality Questionnaire (FQQ), which measures key dimensions of peer friendship (such as companionship, conflict, help/support, security, and closeness) (25). The FQQ will quantify each participant's perception of their friendship, providing insight into the social relationship formed through the intervention.

### Adverse events

Any adverse events in either group will be documented using case report forms, including monitoring for social or emotional distress, with caregiver involvement as needed. The trial coordinator will maintain regular contact with participants (or parents/guardians of participants with DS) to monitor for any issues or adverse effects throughout the study.

### Statistical analysis

A target sample size of 12 dyads (12 per group) was selected to provide preliminary estimates of variability and feasibility, consistent with recommendations for pilot trials (26, 27), while remaining pragmatic given resource constraints in DS population studies. Statistical analysis will be performed by a researcher blinded to group allocation, and an independent statistician will verify the primary outcome analysis. The primary analysis will compare the shared book reading intervention group and the waitlist control group for the primary outcome at 8 weeks post-randomization. Secondary analysis will include analysis of secondary outcomes at 8 weeks post-randomization. Additionally, we will use an actor–partner interdependence model (APIM) to explore within-dyad relationships (examining how one member's baseline quality of life or mental wellbeing might influence both their own and their partner's outcomes). Dyad composition and potential differences in interpersonal dynamics will be accounted for within the APIM. For outcome comparisons, we will use mixed-effects linear models to account for repeated measures and dyad clustering, and results will be presented as mean differences between groups with 95% confidence intervals. Models will adjust for baseline scores in outcomes and covariates, including age, sex, and education level.

The primary analysis will be conducted using an intention-to-treat (ITT) approach. Secondary analyses will be performed after imputation of missing data. A sensitivity analysis will be conducted using multiple imputations for missing data (including all secondary outcomes and covariates in the imputation model). All analyses will be performed using R in RStudio (28).

Feasibility of the RCT will be evaluated based on participant adherence to the intervention and completion rates of outcome measures at each time point. Acceptability of the intervention will be assessed through qualitative feedback from participants in the intervention arm. Caregiver presence and group size will be documented and analyzed as potential moderators of intervention effects. We will maintain continuous consultation with the PPI panel throughout the study to gain insights into intervention implementation. This ongoing feedback will help researchers understand factors that encourage or discourage participation, improving study processes for consideration in future studies. We will also involve the PPI panel in disseminating findings, incorporating their input when presenting results to local stakeholders.

### Data confidentiality

All information related to the trial will be securely stored at the research center of the Faculty of Health Sciences, University of Macau. Data will be anonymized using coded identification numbers. These codes will be stored separately under password protection to prevent identification of individual participants. Access to the data will be limited to a small number of personnel responsible for quality control, auditing, and analysis. The final trial dataset will only be accessible to the study statistician.

## Discussion

### Anticipated contributions

Our study adopted a PPI approach, engaging two parents, one young people with DS, two university students in an iterative consultation process, subsequently joined by one MDSA representative and three additional undergraduates. Initial consultations revealed stakeholder recommendations to utilize social media platforms (e.g., DS-focused Facebook groups) and MDSA newsletters for recruitment, with 85% of caregivers preferring Sunday afternoon sessions to avoid scheduling conflicts with educational/therapeutic commitments. Young participants emphasized incorporating engaging activities (e.g., games) to maintain involvement. Subsequent consultations refined the intervention protocol through: (i) inclusion of visually enriched books (e.g., Unusual Friends) and graphic novels as suggested by PPI members; (ii) co-development of a parent-proposed “growth record” logbook with visual prompts (e.g., emoticons); and (iii) implementation of a 1-h DS-specific communication training for student volunteers covering simplified language and visual supports. Protocol modifications included introducing 10-min icebreaker activities (e.g., adapted musical chairs), structured dyadic reflection periods, replacement of lengthy quality-of-life measures with PedsQL 4.0 (short form), and addition of the FQQ per parental requests. A PPI advisory group (two parents, two DS youth, three students) meets quarterly to monitor adherence (e.g., attendance records) and troubleshoot implementation challenges (e.g., introducing audiobooks for reading difficulties), while contributing to knowledge dissemination through plain-language summaries. While acknowledging potential selection bias (e.g., overrepresentation of highly educated participants), diversity was enhanced through MDSA's outreach initiatives, demonstrating PPI's comprehensive influence across intervention design, implementation, and evaluation.

The goal of this study is to assess the potential feasibility and possible effects of shared book reading as a simple, low-cost intervention to improve the mental wellbeing and quality of life for young people with and without DS. We chose shared book reading because it is an easy-to-implement, broadly applicable activity that enhances participants' overall wellbeing by promoting emotional connections, enhancing social interaction, and providing psychological support. As a pilot study, this research will primarily inform the feasibility of conducting a larger future trial—including acceptability for recruitment, adherence to weekly sessions, and completeness of data collection—and provide initial indications of effect sizes for outcomes of interest. Shared book reading is a non-invasive, low-intensity intervention that requires no complex equipment or professional technical support and can be easily implemented in various settings.

This simplicity makes the shared book reading intervention particularly appealing, with potential for scalability, especially in resource-limited settings for long-term sustained implementation. We anticipate high participant acceptability based on initial PPI feedback indicating positive reception of the concept. Given its non-invasive nature and early positive reception, we believe the intervention will be both feasible and acceptable in practice. While we do not anticipate major implementation challenges, this will be formally evaluated in the pilot study.

### Strengths of the study design

A key strength of our study design is that the shared book reading intervention was developed with in-depth guidance from patients and PPI representatives. Through multiple rounds of discussion, we incorporated perspectives from different stakeholders. For example, caregivers of individuals with DS shared their experiences and expectations for reading activities, public representatives provided feedback on general community acceptance of such programs, and researchers ensured the intervention format was evidence-based and effective. The final intervention protocol represents a truly collaborative product, refined repeatedly through follow-up PPI meetings to maximize the positive benefits of reading and create a comfortable physical and mental environment for participants.

### Addressing gaps in literature

Recent research suggests that interventions aiming to develop language skills in people with DS can indeed improve communication (29). Such interventions can take many forms, including incorporating books and magazines into regular activities and providing adequate guided practice (30). Previous meta-analyses have shown that language interventions, like shared book reading, can improve spoken language ability in children with neurodevelopmental disorders (31). Interventions included in these meta-analyses used various techniques, including shared reading, general language stimulation, direct language instruction, play-based language interventions, multimedia-assisted interventions, social interaction-based interventions, and family-involved interventions. Meta-regression indicated that longer sessions over extended periods were more beneficial than shorter, short-term interventions. This finding informed our decision to implement relatively longer sessions over an 8-week period in our trial, with the expectation that a sustained intervention could lead to larger effect sizes. Although our pilot is not fully powered to detect statistically significant differences, it will provide effect size estimates to inform the sample size of a future definitive RCT.

### Implications for practice and policy

Shared book reading among young people with DS has received less empirical attention than among typically developing adolescents (32). Given the prevalence of language learning difficulties among young people with DS, adjustments in how shared book reading is conducted may be necessary to maximize its effect. In our study, we chose books that combine pictures with text, as evidence suggests these format benefits individuals with DS by compensating for poorer verbal short-term memory through stronger visuospatial memory (33). Lack of motivation and difficulty maintaining focus are commonly reported barriers to reading activities with children with DS (34). Our inclusion of interactive games and the growth logbook in the intervention is partly intended to address these issues by making sessions enjoyable and rewarding. Our pilot will also explore areas that remain unclear in the literature—for example: potential long-term benefits of shared reading; whether effects differ between participants with DS and their student partners; how cultural context might influence outcomes; and the overall sustainability and acceptability of such interventions (35). In summary, this study will yield valuable information for future intervention research. Findings on feasibility and acceptability will guide the design and implementation of larger studies. Ultimately, we aim to establish shared book reading as a viable, evidence-based strategy to improve mental wellbeing and quality of life in young people both with and without DS.

### Potential strengths and limitations

Shared book reading represents a simple, low-cost intervention, and this study constitutes the first attempt to partner young people with DS and university students in a collaborative reading program. This approach is significant because a single intervention targeting both populations may generate sustainable positive effects for both groups. Additionally, this will be the first study conducted in Macau focusing on this specific population, and as such, recruitment rates and expected adherence remain uncertain. The intervention, jointly designed through PPI, demonstrates significant advantages. These findings hold important policy implications, particularly regarding the scalability of this low-cost, community-based model for other neurodevelopmental disorders in resource-limited settings. By demonstrating benefits for both adolescents with disabilities and student partners, it has potential to inform inclusive education policies and provide opportunities for health system integration. Future implementation should focus on promoting the standardized of personnel training and ensuring equitable access to appropriate reading materials and technologies.

However, several limitations should be acknowledged. First, the use of a waitlist control design, rather than an active concurrent control, may introduce potential bias, as control group participants will eventually receive the intervention. This design also raises the possibility of contamination, as control group participants may have access to books and opportunities to engage in shared reading with others outside the study. Second, blinding of participants to their group allocation (intervention or control) is not feasible, which may lead to differing expectations of improvement and influence outcomes during data collection. Finally, challenges related to recruitment and participant retention are anticipated, which could impact the study's overall feasibility and generalizability. As a pilot study, the sample size is limited, and the study may be underpowered to detect modest intervention effects. Consequently, any outcome differences will be interpreted cautiously and primarily used to inform the design of a larger trial. Despite these limitations, this pilot study represents an important first step in evaluating an innovative intervention. The insights gained will be invaluable for planning a definitive trial and could open new avenues for mental wellbeing promotion in young people with developmental disabilities (36).

## Conclusion

This pilot study will inform the design of a subsequent fully powered randomized controlled trial by providing valuable data on the intervention's feasibility, acceptability, and preliminary efficacy. If successful, the shared reading program could ultimately improve the wellbeing of people with DS by providing a new avenue for social engagement and learning. Should the intervention demonstrate improvements in quality of life, it would support the use of shared book reading as an evidence-based, non-pharmacological option to enhance quality of life in the DS community. It may also encourage more programs that connect individuals with DS with other young people (such as university students) in mutually beneficial activities.
