# Supplementary material for: Shared book reading to promote mental well-being among young people with and without Down syndrome: a pilot dyadic randomized controlled trial
Source: Front Public Health. 2025 Oct 29;13:1604241. doi: 10.3389/fpubh.2025.1604241 (PMC12605047; doi:10.3389/fpubh.2025.1604241)
Supplement: Supplementary file 1 [file Supplementary_file_1.docx]

Supplement File 1: Intervention Facilitator Manual

Activity Theme

Share the blue sky and live in harmony

- **Activity objective**
- Promote social interaction
- Reduce feelings of loneliness
- Enhance cognitive understanding
- Improve language expression skills
- **Activity process**

The activity will be held at the community center and last approximately one hour. This event was initiated by Prfo. Rob Smith from the Faculty of Health Sciences at the University of Macau, planned by Huang Xiaoyi, a doctoral student from the Faculty of Health Sciences at the University of Macau, and recruited and assisted by the Macau Down Syndrome Association. Before each intervention activity begins, there will be one or more ice-breaking social games, aiming to involve every child in the activity, which lasts about 10 minutes.

- **Pass the flower while beating the drum**

Game rules: When the first music starts, the first child begins to pass the flower in their hand from left to right to the next child. The volunteer teacher will randomly stop the music based on the children's passing progress. When the music stops, observe which child's hand the flower has been passed to Then the child has to follow the volunteer teacher's instructions to complete a certain task or action (they can participate in the lottery to win a gift).

- **Imitation game**

Game rules: By imitating a series of actions, children will learn happily and enhance their self-expression and body control abilities. Let children exercise their powers of observation, reaction speed and body coordination through imitation. The teacher will stand in front of the children as a demonstration of the movements. The teacher will demonstrate a series of movements, including but not limited to clapping hands, stomping feet, and spinning in circles. The children need to concentrate, carefully observe the teacher's movements, and imitate them as accurately as possible. The accuracy and fluency of action imitation will be used as the scoring criteria, and the teacher will give immediate feedback and encouragement based on the children's performance.

- **Shared book reading**

This meeting will launch a book-sharing reading activity. The specific arrangements are as follows:

At the beginning of the meeting, we will introduce a book-sharing reading activity, aiming to promote interaction and learning among participants. We specially invite participants to take part in the event in pairs and choose one of the 20 books we have carefully selected to read together. These books were selected in collaboration with various stakeholders before the research began, covering a wide range of age groups from preschool education to adolescents and diverse topics. Each pair of participants can freely decide how to share their reading experience. Although we encourage active participation in reading, there are no rigid regulations on the specific sharing methods, allowing participants to arrange according to their own preferences. For example, participants can choose to read aloud, take turns reading, or assign roles to read the dialogue, in order to increase the interactivity and interest of reading. If they wish, two members of the intervention group can also join the group and share the joy of reading with other participants. To ensure the smooth running of the event, several graduate student counselors will coordinate with each pair of participants, supervise the reading process, and ensure that all participants can fully engage in the event. In addition, if caregivers are interested, they are also welcome to join the reading activities and enjoy the reading time with the children. Through such an arrangement, we expect that each participant can find pleasure in shared reading, while promoting the exchange of knowledge and personal growth.

The last 10 minutes of each session will be dedicated to sharing and reflection activities. This session aims to provide a platform for participants to have in-depth exchanges of their experiences and insights during the reading process. Participants will have the opportunity to share what they have read, which not only includes summaries of books but also their personal understanding and feelings. The sharing and reflection activities encourage participants to raise questions and discuss and answer the sharing of other participants, thereby promoting the exchange of knowledge and the collision of ideas. In addition, this session is also a crucial moment for planning the reading goals and plans for the next stage, helping participants clarify the direction of their subsequent reading. Sharing and reflection activities are not only platforms for the exchange of knowledge and information, but also opportunities for participants to build social connections. Especially for the duo, this is a precious time to enhance mutual understanding and cooperation. With such an arrangement, we expect each session to come to a successful conclusion through in-depth sharing and reflection, while laying a solid foundation for social interaction among participants and future reading plans.

- **Growth book**

The research team plans to use growth record books as part of the study to track and document the progress and growth of participants during shared reading activities. Each participant will receive a dedicated growth log to detail their reading journey, the books they have read, the new vocabulary they have learned, and any significant progress they have observed in the reading plan. We encourage caregivers to actively participate in the recording process, which will enable them to have a more intuitive understanding of the development of the activity and continue to support and inspire the patient's reading activities in the family environment. The growth log will serve as a tool for participants to review and celebrate their personal achievements, enhancing their motivation to continue participating in research. In addition, the growth record book will serve as a communication bridge among participants, caregivers and college students, promoting their sharing of reading experiences and feelings, and deepening mutual understanding and connection. Through such measures, we expect that the growth record book can not only enhance the participation of participants, but also stimulate the interest and support of caregivers, while providing a shared and communicative platform for all relevant parties to jointly promote the in-depth development of reading activities.

Supplement File 2: Fidelity Checklist and Scoring Criteria

| Criteria | Yes/No | Scoring |
| --- | --- | --- |
| Icebreaker activity completed (10 min) | ☐ | 1 point |
| Reading blocks with stretch breaks | ☐ | 1 point |
| Visual prompts (emoji cards) used | ☐ | 1 point |
| Logbook entries documented | ☐ | 1 point |
| Facilitator followed pacing guide | ☐ | 1 point |

Fidelity was monitored using a 5-item checklist (see Supplement File 2). Sessions achieving ≥80% of criteria (4/5 points) were deemed adherent.

Supplement File 3: Participant Growth Logbook Template

*Used in the intervention for recording shared reading sessions (handwritten or printed).*

Basic Information

Start Date: __________

Session Log Table

| Date | Book Title | New Words | Favorite Part (Emoji) | Caregiver Notes |
| --- | --- | --- | --- | --- |
| 08/02/2025 | *Unusual Friends* | “Confidence”  “Friendship” | 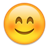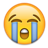 | Enjoyed the books.  Loved discussing the pictures |
|  |  |  |  |  |
|  |  |  |  |  |
|  |  |  |  |  |
|  |  |  |  |  |
